# KDM5B promotes self‐renewal of hepatocellular carcinoma cells through the microRNA‐448–mediated YTHDF3/ITGA6 axis

**DOI:** 10.1111/jcmm.16342

**Published:** 2021-04-07

**Authors:** Jun‐Cheng Guo, Zhuo Liu, Yi‐Jun Yang, Min Guo, Jian‐Quan Zhang, Jin‐Fang Zheng

**Affiliations:** ^1^ Hainan General Hospital Haikou China; ^2^ Hainan Medical University of Hainan Hospital affiliated Haikou China; ^3^ Central South University Xiangya School of Medicine Affiliated Haikou Hospital Haikou China

**Keywords:** demethylation, hepatocellular carcinoma, integrin subunit alpha 6, lysine‐specific demethylase 5B, m6A modification, microRNA‐448, self‐renewal, YTH N6‐methyladenosine RNA binding protein 3

## Abstract

Histone methylation plays important roles in mediating the onset and progression of various cancers, and lysine‐specific demethylase 5B (KDM5B), as a histone demethylase, is reported to be an oncogene in hepatocellular carcinoma (HCC). However, the mechanism underlying its tumorigenesis remains undefined. Hence, we explored the regulatory role of KDM5B in HCC cells, aiming to identify novel therapeutic targets for HCC. Gene Expression Omnibus database and StarBase were used to predict important regulatory pathways related to HCC. Then, the expression of KDM5B and microRNA‐448 (miR‐448) in HCC tissues was detected by RT‐qPCR and Western blot analysis. The correlation between KDM5B and miR‐448 expression was analysed by Pearson's correlation coefficient and ChIP experiments, and the targeting of YTH N6‐methyladenosine RNA binding protein 3 (YTHDF3) by miR‐448 was examined by luciferase assay. Additionally, the effect of KDM5B on the proliferation, migration, invasion and apoptosis as well as tumorigenicity of transfected cells was assessed using ectopic expression and depletion experiments. KDM5B was highly expressed in HCC cells and was inversely related to miR‐448 expression. KDM5B demethylated H3K4me3 on the miR‐448 promoter and thereby inhibited the expression of miR‐448, which in turn targeted YTHDF3 and integrin subunit alpha 6 (ITGA6) to promote the malignant phenotype of HCC. Moreover, KDM5B accelerated HCC progression in nude mice *via* the miR‐448/YTHDF3/ITGA6 axis. Our study uncovered that KDM5B regulates the YTHDF3/ITGA6 axis by inhibiting the expression of miR‐448 to promote the occurrence of HCC.

## 1 INTRODUCTION

Hepatocellular carcinoma (HCC) is the leading type (~90%) of primary liver cancer, having an incidence of about 850 000 cases every year and causing many deaths worldwide.^1^, ^2^ Chronic HBV/HCV infection, alcohol, cigarette smoking, fatty liver and diabetes are all risk factors for HCC.^3^ Most HCC patients are diagnosed at an advanced stage, and only 30% of HCC cases can be treated by resection, leading to dismal prognosis.^4^ In addition to supporting early diagnosis, the advent of novel molecular biomarkers could improve the survival rates of the patients with advanced HCC.^5^ For instance, MYB proto‐oncogene like 2 and lysine‐specific demethylase 5B (KDM5B) regulate hub genes obtained *via* the Gene Expression Profiling Interactive Analysis (GEPIA) database that are related to the poor prognosis of patients with HCC.^6^ However, the underlying molecular mechanisms of the HCC progression still have not been fully elucidated,^7^ which drew our attention to explore novel therapies targeting HCC‐specific molecular disorders for patients with HCC.^8^

KDM5B is a histone demethylase contained in the JmjC domain, which is capable of demethylating tri‐ and dimethyl modifications of H3 lysine 4.^9^ It is overexpressed in multiple cancers, including stomach cancer, glioma and breast cancer.^10^ KDM5B has emerged as a cancerogenic factor in HCC, as demonstrated by promoted HCC cell proliferation and colony formation.^11^ In addition, microRNA‐448 (miR‐448) has been reported to have low expression in HCC samples and to delay epithelial‐mesenchymal transition and invasion in HCC cells by down‐regulating rho‐associated kinase 2 (ROCK2).^12^ Accumulating evidence indicates that KDM5B knockdown could increase miR‐448 expression to impede the growth of papillary thyroid cancer cells.^13^ Furthermore, a bioinformatic analysis in our present study suggests that miR‐448 may target YTH N6‐methyladenosine RNA binding protein 3 (YTHDF3). YTHDF3 is known as a member of the readers of RNA methylation (m6A) family, and it is reported to be highly expressed in HCC cells.^14^, ^15^ Hence, we speculated that the demethylase KDM5B may inhibit the expression of miR‐448 to mediate YTHDF3, thus contributing to the occurrence of HCC. In support of this hypothesis, we transfected HCC cells with a series of mimics, inhibitors and short hairpin RNAs (shRNAs) to analyse in detail the potential molecular mechanisms regarding the KDM5B/miR‐448/YTHDF3 axis in HCC development.

## 2 MATERIALS AND METHODS

### 2.1 Tissue sample collection

Twelve pairs of fresh HCC tissues and corresponding normal tissues were obtained from patients with HCC. The fresh tissues were frozen by immersion in liquid nitrogen for later mRNA analysis. Enrolled patients had no previous radiotherapy or chemotherapy history and were followed up until death or the end of the study, which ranged from 3 to 59 months. All samples used in this study underwent histopathological examination.

### 2.2 Bioinformatic analysis

HCC‐related differentially expressed genes were identified after differential analysis of the Gene Expression Omnibus (GEO) microarray database (https://www.ncbi.nlm.nih.gov/gds) including GSE45267, GSE62232 and GSE117361 using the R package limma (http://www.bioconductor.org/packages/release/bioc/html/limma.html) (Table 1), as well as differential analysis of the Liver Hepatocellular Carcinoma (LIHC) data set in The Cancer Genome Atlas (TCGA) (https://portal.gdc.cancer.gov) using GEPIA (http://gepia2.cancer‐pku.cn/#index). Human transcription factors were obtained from Cistrome (http://cistrome.org). Then, a Venn diagram was drawn for the intersection of differentially expressed genes and transcription factors. The key transcription factor was determined to be KDM5B on the basis of existing literature (difference analysis threshold: |logFC| > 0.5, *P* < .05). The ChIPBase database (http://rna.sysu.edu.cn/chipbase/index.php) suggested that KDM5B binds to the miR‐448 promoter. Subsequently, the key downstream gene of miR‐448 was predicted *via* the TargetScan (http://www.targetscan.org/vert_71/) (cumulative weighted context ++ score < −0.05, total context ++ score < −0.05), StarBase (http://starbase.sysu.edu.cn/) (clipExpNum > 15, pancancerNum > 1), mirDIP (http://ophid. utoronto.ca/mirDIP/) (integrated score > 0.55), miRDB (http://www.mirdb.org) (target score ≥ 60) and microRNA databases (http://www.microrna.org/) (conservation > 0.75, energy < −10, mirsvr_score < −0.2), and was identified through intersection in the Venn diagram. Further, the key downstream gene was determined from the existing literature to be YTHDF3. The microRNA database was used to obtain binding sites between miR‐448 and YTHDF3, and StarBase was applied to determine the expression profile of YTHDF3 and integrin subunit alpha 6 (ITGA6) and the correlation of their expression in HCC. Multi Experiment Matrix (MEM, https://biit.cs.ut.ee/mem/index.cgi) was adopted to detect the possible co‐expression relationship between YTHDF3 and ITGA6.

**TABLE 1:** Information on GEO microarray data sets

GSE# ID	Platforms	Sample size	Normal	Tumour	Postscript
GSE45267	GPL570	32	17	15	Deleting 55 unrelated samples
GSE62232	GPL570	91	10	81	log2 processing of data
GSE117361	GPL6480	4	2	2	log2 processing of data

### 2.3 Cell culture and transfection

Hep3B, SMMC7721 and HEK293T cells were purchased from American Type Culture Collection (ATCC) and cultured with Roswell Park Memorial Institute (RPMI)‐1640 medium (Gibco Company) containing 10% foetal bovine serum (FBS) (Gibco), 10 μg/mL streptomycin and 100 U/mL penicillin in an incubator (Thermo Fisher Scientific Inc) at 37°C with 5% CO_2_. HCC cells in the logarithmic growth phase were seeded into 6‐well culture plates at a density of 4 × 10^5^ cells/well. When reaching 80%‐90% confluence, the cells were transfected with miR‐448 mimic and miR‐448 inhibitor as well as their corresponding negative controls (NCs) (mimic NC and inhibitor NC) according to the instructions of the Lipofectamine 2000 reagents (11668‐019; Invitrogen). The sequences were designed by and plasmids purchased from Shanghai GenePharma Co, Ltd.

HEK293T cells were transfected with KDM5B overexpression vector (oe‐KDM5B), YTHDF3 overexpression vector (oe‐YTHDF3), ITGA6 overexpression vector (oe‐ITGA6) or NC of overexpression vector (oe‐NC) produced by Shanghai GenePharma Co, Ltd., through the packaging virus and the target vector, and the supernatant was collected after 48 hours of culture. Exponentially replicating viruses in the supernatant were collected, and the cells were infected with oe‐NC, oe‐KDM5B, oe‐YTHDF3, oe‐ITGA6 and oe‐KDM5B + sh‐ITGA6. Then, the cells in the logarithmic growth phase were detached with trypsin and isolated to obtain suspension of 5 × 10^4^ cells/mL, which was seeded in a 6‐well plate (2 mL per well) for incubation at 37°C overnight.

### 2.4 Reverse transcription quantitative polymerase chain reaction (RT‐qPCR)

Total RNA was extracted using TRIzol reagents (15596026; Invitrogen) and reversely transcribed into complementary DNA (cDNA) according to the instructions of PrimeScript RT reagent Kit (RR047A; Takara Bio Inc). RT‐qPCR was performed on the synthesized cDNA using the Fast SYBR Green PCR kit (Applied Biosystems Inc) and an ABI PRISM 7300 RT‐qPCR system (Applied Biosystems). The relative expression of mRNA or miRNA was normalized to glyceraldehyde‐3‐phosphate dehydrogenase (GAPDH) or U6, respectively, and was calculated using the 2^−ΔΔCt^ method. The primer design is shown in Table 2.

**TABLE 2:** Primer sequences

	Sequences
KDM5B	F: 5′‐TTCCACAGCTTGCTGAGATG‐3′
	R: 5′‐GCCATAGCTTTCTCCACTGC‐3′
GAPDH	F: 5′‐GTGGACCTGACCTGCCGTCT‐3′
	R: 5′‐GGAGGAGTGGGTGTCGCTGT‐3′
miR‐448	F: 5′‐TTATTGCGATGTGTTCCTTATG‐3′
	R: 5′‐ATGCATGCCACGGGCATATACACT‐3′
U6	F: 5′‐CGCTTCGGCAGCACATATACTAAAATTGGAAC‐3′
	R: 5′‐GCTTCACGAATTTGCGTGTCATCCTTGC‐3′
ITGA6	F: 5′‐GGCGGTGTTATGTCCTGAGTC‐3′
	R: 5′‐AATCGCCCATCACAAAAGCTC‐3′
YTHDF3	F: 5′‐TCAGAGTAACAGCTATCCACCA‐3′
	R: 5′‐GGTTGTCAGATATGGCATAGGCT‐3′

### 2.5 Western blot analysis

Cells from each culture group were detached by trypsin, collected, and lysed with an enhanced radioimmunoprecipitation assay lysis buffer (Wuhan Boster Biological Technology Co., Ltd.) containing a protease inhibitor. After measurement of protein concentration with a bicinchoninic acid (BCA) quantitation kit (Boster Biological Technology), samples of protein were separated by 10% sodium dodecyl sulphate‐polyacrylamide gel electrophoresis, and electrotransferred onto a polyvinylidene fluoride membrane. The membrane was blocked in 5% bovine serum albumin (BSA) at room temperature for 1 hour and added with diluted primary antibodies (antibody to KDM5B, ab181089; Abcam; antibody to YTHDF3, Cat # 25537‐1‐AP; Proteintech; antibody to ITGA6, Cat # 3750; CST) for incubation overnight at 4°C. The following day, the membrane was washed 3 times with Tris‐buffered saline Tween‐20, re‐probed with horseradish peroxidase (HRP)‐labelled secondary antibody of goat anti‐rabbit for 1 hour at room temperature and developed with enhanced chemiluminescence working solution (EMD Millipore). Finally, ImageJ analysis software was used to quantify the grey levels of each band in the Western blot image normalized to GAPDH.

### 2.6 Immunohistochemistry

The streptavidin peroxidase (SP) method was used for this experiment according to the instructions of an immunohistochemistry kit (SP0041; Beijing Solarbio Science & Technology Co., Ltd.). Staining was scored by two independent pathologists. The stained area was defined as the percentage of positive stained tissues in the whole tissue area, and the scoring criteria were as follows: 0, <10%; 1, 10%‐25%; 2, 26%‐50%; 3, 50%‐75%; and 4, >75%. The staining intensity was scored as 0‐3 (negative: 0 point; weak expression: 1 point; positive: 2 points; strong positive: 3 points). The final staining score of KDM5B/YTDF3/ITGA6 was the sum of staining intensity and staining degree. For statistical analysis, final staining scores of 0‐5 indicated low expression and final staining scores of 6‐7 suggested high expression.

### 2.7 Dual‐luciferase reporter gene assay

The dual‐luciferase reporter gene vectors for 3′ untranslated region (UTR) of YTHDF3 and mutant plasmid of YTHDF3 with mutations in the binding sites with miR‐448 were constructed: pmirGLO‐YTHDF3‐WT and pmirGLO‐YTDHF3‐MUT. The reporter plasmids were cotransfected with miR‐448 mimic and the NC plasmid into HEK293T cells, which were lysed 24 hours after transfection and centrifuged at 6037 *g* for 1 minute, with collection of the supernatant. The Dual‐Luciferase^®^ Reporter Assay System (E1910; Promega) was used to detect luciferase activity. A total of 100 μL Firefly luciferase working fluid and 100 μL Renilla luciferase working fluid were added into each cell sample with the Renilla luciferase as the internal standard, and the ratio of Firefly luciferase activity to Renilla luciferase activity represented the relative luciferase activity.

### 2.8 Spheroid formation assay

The HCC cells of each group in logarithmic growth phase were evenly spread on a 6‐well ultra‐low adhesion culture plate (1 × 10^3^ cells/mL; Corning Glass Works) and were cultured in serum‐free Dulbecco's modified Eagle's medium/Ham's F‐12 (DMEM/F12) medium containing 2 mmol/L l‐glutamine, 1% sodium pyruvate, 100 μg/mL penicillin G, 100 μg/mL streptomycin, 20 ng/mL epidermal growth factor, 10 ng/mL fibroblasts growth factor‐2 (FGFR‐2), N2 and B27 (all purchased from Invitrogen) for 1‐2 weeks. Finally, the number of tumour sphere cells larger than 50 μm in diameter was counted under a stereomicroscope.

### 2.9 Agarose colony formation assay

HCC cells of each group in the logarithmic growth phase were suspended in the culture medium and 1 mL 0.5% agarose was added to a 6‐well plate and solidified at room temperature to prepare the bottom gel. Then, 500 μL cell suspension containing 5000 cells was mixed with 500 μL 0.5% agarose to prepare the upper gel, which was placed on the bottom and allowed to solidify at room temperature. After coagulation, the sample was added into a 2 mL medium and cultured at 37°C with 5% CO_2_ for 3 weeks. Cells were fixed with ethanol, stained with 0.1% crystal violet (C0004; Banmanbio Co, Ltd.) and then imaged with a microscope. The number of clones was counted with ImageJ software.

### 2.10 Scratch test

Horizontal lines were drawn with a ruler and a marker at intervals of 0.5‐1 cm on the bottom surface of a 6‐well plate, with at least five lines passing through each well. Cells were added to the 6‐well plate at a density of approximately 5 × 10^5^ cells/well and were incubated overnight in a medium containing 10% FBS. A sterile 10 μL pipette was used to make scratches perpendicular to the horizontal lines. The length of the wounds was measured under an optical microscope at 0 and 24 hours incubation, and images were collected under an inverted microscope to observe the cell migration in each group.

### 2.11 Transwell assay

The apical chamber surface of the bottom membrane of the Transwell chamber was coated with Matrigel (BD Biosciences) which was polymerized into a gel at 37°C for 30 minutes, with hydration of the basal membrane before use. The cells were cultured in serum‐free medium for 12 hours, followed by collection and resuspension of cells in serum‐free medium (1 × 10^5^/mL). The lower chamber was added with 10% FBS, and 100 μL cell suspension was added into the Transwell chamber at 37°C for 24 hours. The cells that had not invaded the Matrigel membrane surface were gently removed with a cotton swab, and the remaining cells were fixed with 100% methanol and stained with 1% toluidine blue (Sigma‐Aldrich Chemical Company). Stained invading cells in five randomly selected areas were manually counted under an inverted light microscope (Carl Zeiss).

### 2.12 Chromatin immunoprecipitation assay (ChIP)

ChIP assay was performed using a ChIP kit (Millipore). After reaching 70%‐80% confluence, cells collected from each group were added with 1% formaldehyde and fixed at room temperature for 10 minutes to induce DNA‐protein cross‐linking. Then, the cells were subjected to ultrasonic treatment to produce fragments of appropriate size. The fragments were centrifuged at 6540 *g* at 4°C with the supernatant collected into three tubes which were added with positive control antibody to RNA polymerase II, as well as NC antibodies to human immunoglobulin G and KDM5B (ab181089; Abcam) or H3K4me3 (ab12209; Abcam) for incubation at 4°C overnight. Subsequently, Protein Agarose/Sepharose was used to precipitate the endogenous DNA‐protein complex, with the supernatant discarded after centrifugation. The non‐specific complexes were washed, and de‐cross‐linking was performed following incubation at 65°C overnight. Afterwards, phenol/chloroform was added to purify and recover DNA fragments. Finally, RT‐qPCR was performed to examine the expression of miR‐448.

### 2.13 Xenograft tumour in nude mice

Thirty female BALB/c nude mice aged 3‐4 weeks were purchased from Beijing Institute of Pharmacology, Chinese Academy of Medical Sciences, fed in a specific pathogen‐free animal laboratory in separate cages with humidity of 60%‐65% and a temperature of 22‐25°C, and provided with free access to food and water under a 12‐hour light/dark cycle. The experiment was started one week after adaptive feeding, before which the health status of mice was observed. The mice were randomly arranged into six groups according to the bodyweight with 5 mice in each group. HCC cells (5 × 10^6^ cells/mouse) that had been transfected with oe‐NC + sh‐NC, oe‐KDM5B + sh‐NC, or oe‐KDM5B + sh‐ITGA6, or treated with NS, GSK‐467 (a selective inhibitor of KDM5B) + oe‐NC or GSK‐467 + oe‐ITGA6 were then subcutaneously implanted into the back of mice. The status of nude mice was monitored following the procedure, and tumour formation and growth were evaluated weekly. After 4 weeks, the mice were killed under deep anaesthesia, tumours were removed, and their weight and volume were measured after back tumour was stripped, and the expression of miR‐448 and ITGA6 was measured using RT‐qPCR.

### 2.14 Statistical analysis

The SPSS 21.0 version (IBM Corp.) was used for statistical analysis. Measurement data were presented as mean ± standard deviation. The data of cancer tissues and corresponding normal tissues were analysed by paired *t* test, and the data of the other two groups were analysed by unpaired *t* tests. Besides, the data among multiple groups were analysed by one‐way analysis of variance (ANOVA) and Tukey's post hoc test, the data among groups at different time were analysed by repeated measures ANOVA followed by Bonferroni's post hoc test. Pearson's correlation coefficient was used to assess the correlation of indicators. Statistical significance was assumed when *P* < .05.

## 3 RESULTS

### 3.1 KDM5B deletion inhibited self‐renewal of HCC cells

**FIGURE 1:**
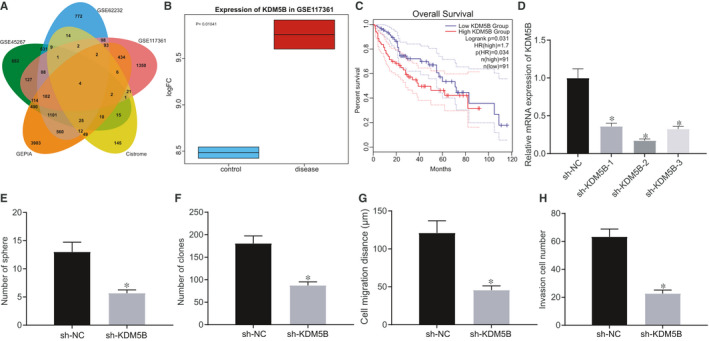
KDM5B deletion suppresses the self‐renewal of Hep3B cells. A, Venn diagram displaying the intersection of differentially expressed genes (SOX4, SMARCC1, KDM5B and JUNB) obtained from analysing the GSE45267, GSE62232 and GSE117361 microarray data sets and differentially expressed genes of HCC through GEPIA with human transcription factors obtained from the Cistrome database. B, Box plot of KDM5B expression in microarray data set GSE117361, wherein the left blue boxes indicate the expression in normal samples, and the right red boxes represent the expression in HCC samples. C, Relation between survival curve of patients with HCC and KDM5B expression obtained by GEPIA analysis. D, KDM5B expression in Hep3B cells after sh‐KDM5B‐1, sh‐KDM5B‐2 and sh‐KDM5B‐3 treatment assessed by RT‐qPCR. E, Formation of spheroids after sh‐KDM5B treatment assessed by spheroid formation assay. F, Colony formation ability after sh‐KDM5B treatment assessed by colony formation assay. G, Cell migration after sh‐KDM5B treatment assessed by scratch test. H, Cell invasion after sh‐KDM5B treatment assessed by Transwell assay. The data were measurement data, which were presented as mean ± standard deviation. The data in two groups were compared using unpaired *t* test, and the data in multiple groups were compared using one‐way ANOVA and Tukey's post hoc test. *P < .05 compared with treatment of sh‐NC

A total of 3482, 3594 and 2525 differentially expressed genes were obtained after differential analysis of the microarray data sets GSE45267, GSE62232 and GSE117361, respectively, by R language. GEPIA analysis also obtained 6,987 LIHC differentially expressed genes in the TCGA database. In addition, 318 human transcription factors were obtained from Cistrome, and 4 important transcription factors (SOX4, SMARCC1, KDM5B and JUNB) in HCC were identified by taking the intersection of differentially expressed genes and transcription factors (Figure 1A). A previous study has shown that KDM5B is related to HCC and shows a significantly increased expression in HCC tissue.^11^ By extracting the expression data of the microarray data set GSE117361, we further confirmed that KDM5B is highly expressed in HCC (Figure 1B). GEPIA analysis suggested that its high expression predicted significantly lower survival of HCC patients (Figure 1C), but the mechanism by which it regulates the occurrence of HCC remains uncharacterized. In order to study the effect of KDM5B on the growth of HCC cells, we established a KDM5B knockdown cell line through performing lentivirus infection of the Hep3B cells. Then, RT‐qPCR was conducted to measure the expression of KDM5B in each group of cells. Compared with NC treatment, treatments with sh‐KDM5B‐1, sh‐KDM5B‐2 and sh‐KDM5B‐3 led to significantly lower expression of KDM5B, among which sh‐KDM5B‐2 led to the lowest KDM5B expression (*P* < .05) (Figure 1D), such that the sh‐KDM5B‐2 was used to silence KDM5B in subsequent experiments. After constructing a cell line with low expression of KDM5B, we performed a series of tests to investigate the effect of KDM5B on HCC cell functions. Spheroid formation assay, agarose colony formation assay, scratch test and Transwell assay showed that KDM5B knockdown reduced the ability of cells to form spheroids, weakened the ability of agarose colony formation and impeded the cell migration and invasion (Figure 1E‐H). We further performed the above‐mentioned experiments in the SMMC7721 cell line (Figure S1A‐D), obtaining results consistent with those seen in Hep3B cells. Altogether, KDM5B silencing could inhibit spheroid formation, colony formation, invasion and migration of HCC cells.

### 3.2 KDM5B inhibited miR‐448 by demethylating H3K4me3

**FIGURE 2:**
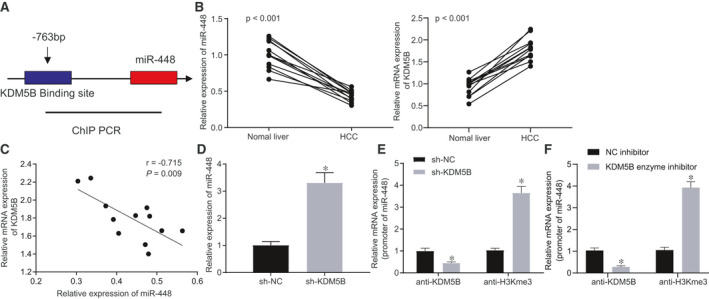
KDM5B inhibits miR‐448 by H3K4me3 demethylation. A, ChIPBase database predicting a possible binding site for KDM5B at 763 bp upstream of miR‐448. B, Relative miR‐448 and KDM5B expression in HCC tissues and corresponding normal tissues. C, Linear relationship between KDM5B and miR‐448 expression in HCC tissues. D, miR‐448 expression in cells after sh‐KDM5B treatment assessed by RT‐qPCR. E, ChIP results after sh‐KDM5B treatment assessed by RT‐qPCR. F, ChIP experimental validation after treatment with inhibitors targeting KDM5B catalytic region. The data were measurement data, which were presented as mean ± standard deviation. The data of tumour tissues and corresponding normal tissues were compared using unpaired *t* test, and the data in the other two groups were compared using unpaired *t* test. Pearson's correlation coefficient was used to assess the correlation of indicators. *P < .05 when compared with treatment of sh‐NC

KDM5B is a transcription repressor, which contains histone demethylase active sites.^16^ Based on the demethylase activity of KDM5B, we searched the ChIPBase database for targets that might be regulated by KDM5B. The results showed a potential KDM5B binding site at 763 bp upstream of miR‐448 (Figure 2A). It has been reported that miR‐448 inhibits HCC by inhibiting the self‐renewal of HCC cells.^12^ We measured the expression of KDM5B and miR‐448 using RT‐qPCR in 12 HCC tissues and 12 corresponding normal tissues and analysed their correlation. The results showed that miR‐448 had low expression in HCC tissues (Figure 2B) and was negatively correlated with the KDM5B expression (Figure 2C). Next, we examined the expression of miR‐448 in Hep3B (sh‐NC) and Hep3B (sh‐KDM5B) cell lines using RT‐qPCR. The results indicated that miR‐448 was distinctly up‐regulated in the sh‐KDM5B–treated Hep3B cell line (Figure 2D). In order to investigate whether the demethylase activity of KDM5B directly inhibited the expression of miR‐448, we first performed ChIP analysis of the miR‐448 promoter with an antibody to KDM5B. RT‐qPCR results suggested that KDM5B knockdown significantly reduced the binding region content of the miR‐448 promoter. Then, we performed ChIP analysis of the miR‐448 promoter with an antibody against H3K4me3. We observed that the miR‐448 promoter binding region was significantly enriched in the sh‐KDM5B–treated Hep3B cells (Figure 2E). Meanwhile, we conducted ChIP experiments in Hep3B cells treated with inhibitors targeting the activity of the KDM5B catalytic region. Results showed that the miR‐448 promoter region was also significantly enriched in KDM5B enzyme inhibitor‐treated cells compared with the control group (Figure 2F), suggesting that the binding of KDM5B to the upstream region of miR‐448 resulted in the loss of H3K4me3. We repeated the above experiments in the HCC cell line SMMC7721 (Figure S1E‐G), which had similar results to those in Hep3B cells. Taken together, KDM5B could directly inhibit the expression of miR‐448 in HCC cells by promoting demethylation of H3K4me3.

### 3.3 miR‐448 inhibited the YTHDF3/ITGA6 axis

**FIGURE 3:**
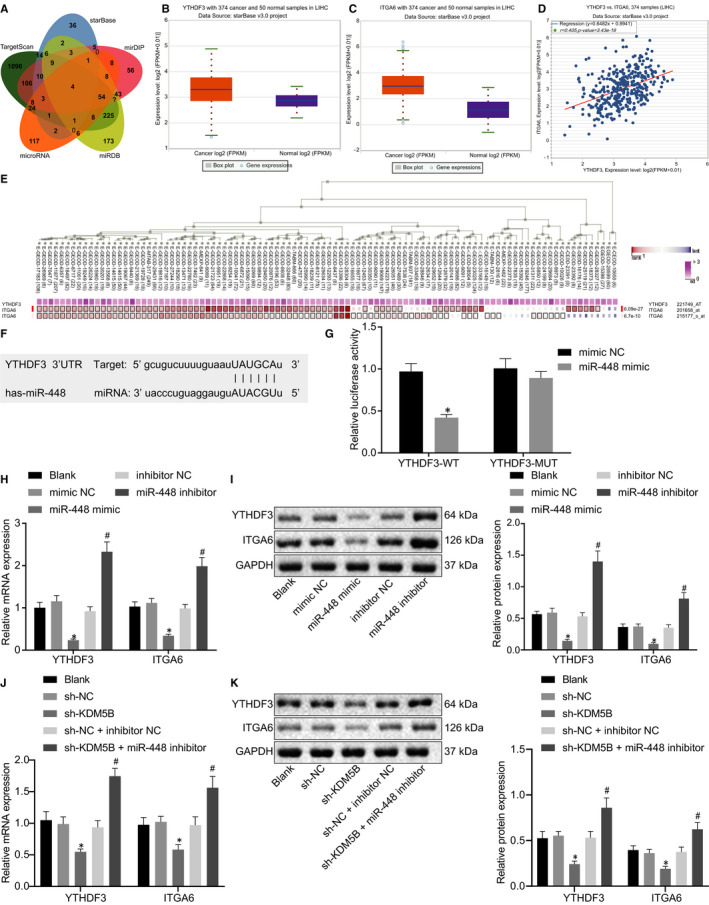
miR‐448 targets YTHDF3/ITGA6 axis. A, Venn diagram predicting downstream genes of miR‐448 based on TargetScan, StarBase, mirDIP, miRDB and microRNA. B, Box plot of YTHDF3 expression after obtained by StarBase, wherein the left red boxes indicate its expression in HCC samples, and the right purple boxes represent its expression in normal samples. C, Box plot of ITGA6 expression obtained by StarBase, wherein the left red boxes represent its expression in HCC samples, and the right purple boxes represent its expression in normal samples. D, Expression correlation diagram between YTHDF3 and ITGA6 in HCC obtained by StarBase. E, MEM analysis displaying a significant co‐expression relationship between YTHDF3 and ITGA6. F, Binding sites between miR‐448 and YTHDF3. G, Luciferase activity after cotransfection of miR‐448 mimic and pmirGLO‐YTHDF3‐WT or pmirGLO‐YTHDF3‐MUT relative to Renilla luciferase activity. H, RT‐qPCR determination of mRNA level of YTHDF3 and ITGA6 in HCC cells after miR‐448 mimic and miR‐448 inhibitor treatment. I, Western blot analysis of the protein level of YTHDF3 and ITGA6 in HCC cells normalized to GAPDH after miR‐448 mimic and miR‐448 inhibitor treatment. J, Detection of YTHDF3 and ITGA6 mRNA expression in HCC cells after cotransfection of sh‐KDM5B and miR‐448 inhibitor by RT‐qPCR. K, Detection of YTHDF3 and ITGA6 protein expression in HCC cells after cotransfection of sh‐KDM5B and miR‐448 inhibitor by Western blot analysis. The data were measurement data, which were presented as mean ± standard deviation. The data in two groups were compared using unpaired *t* test, and the data in multiple groups were compared using one‐way ANOVA and Tukey's post hoc test. *P < .05 when compared with treatment of sh‐NC

TargetScan, StarBase, mirDIP, miRDB and microRNA were used to predict the downstream genes of miR‐448, which yielded 1802, 97, 551, 776 and 245 downstream genes, respectively. Four important downstream genes were obtained by Venn intersection (Figure 3A). Previous studies have shown that YTHDF3 and ITGA6 are both highly expressed in HCC tissues.^15^, ^17^ We predicted through microRNA analysis that the 3′UTR of YTHDF3 may contain a site that binds to miR‐448, and determined that YTHDF3 and ITGA6 were highly expressed in HCC by StarBase analysis (Figure 3B,C), showing significant positive correlation (Figure 3D). MEM prediction also showed significant co‐expression of YTHDF3 and ITGA6 (Figure 3E). Based on the above predictions, we speculated that miR‐448 could target YTHDF3 and inhibit the YTHDF3/ITGA6 axis, thereby inhibiting the occurrence of HCC.

As shown in Figure 3F, the 3′UTR of YTHDF3 contains a possible binding site to miR‐448. In order to verify this, we performed a luciferase assay. For this, we cloned WT and MUT 3′UTR of YTHDF3 into the pmirGLO plasmid, and cotransfected miR‐448 mimic or NC mimic with YTHDF3‐WT or YTHDF3‐MUT into HEK293T cells. The results showed that the luciferase activity was significantly reduced after cotransfection with miR‐488 and YTHDF3‐WT but was not affected when cotransfected with YTHDF3‐MUT, suggesting that miR‐448 could indeed bind to YTHDF3 (Figure 3G). In order to further verify the inhibitory effect of miR‐448 on the YTHDF3/ITGA6 axis, we observed changes in the expression of YTHDF3 and ITGA6 upon overexpressing/inhibiting miR‐448 expression. RT‐qPCR results indicated that overexpressed miR‐448 could significantly inhibit the mRNA level of YTHDF3 and ITGA6. When miR‐448 was inhibited, YTHDF3 and ITGA6 mRNAs were elevated to varying degrees (Figure 3H), which was confirmed by Western blot analysis (Figure 3I). Next, we cotransfected sh‐KDM5B and miR‐448 inhibitor into cells and found that the altered YTHDF3 and ITGA6 expression caused by the sh‐KDM5B treatment alone was rescued after cotransfection of sh‐KDM5B and miR‐448 inhibitor (Figure 3J,K), demonstrating that KDM5B affected downstream molecular pathways by regulating miR‐448. Therefore, miR‐448 targets the YTHDF3 and to suppress the YTHDF3/ITGA6 axis.

### 3.4 miR‐448 inhibited self‐renewal of HCC cells by inhibiting YTHDF3/ITGA6 axis

**FIGURE 4:**
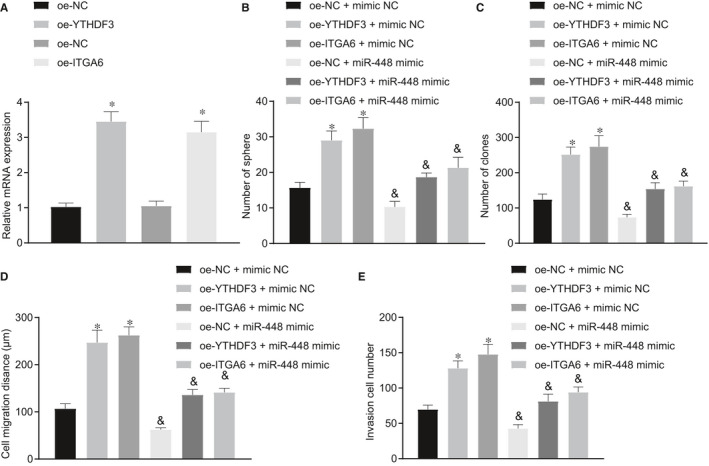
miR‐448 inhibits self‐renewal of HCC cells by suppressing the YTHDF3/ITGA6 axis. A, RT‐qPCR determination of YTHDF3 and ITGA6 expression in Hep3B cells after oe‐YTHDF3 and oe‐ITGA6 treatment. B, Formation of cell spheroids after oe‐YTHDF3, oe‐ITGA6 and miR‐448 mimic treatment. C, Colony‐forming ability assessed by the colony formation assay after oe‐YTHDF3, oe‐ITGA6 and miR‐448 mimic treatment. D, Migratory capacity assessed by the scratch test after oe‐YTHDF3, oe‐ITGA6 and miR‐448 mimic treatment. E, Invasive ability assessed by the Transwell assay after oe‐YTHDF3, oe‐ITGA6 and miR‐448 mimic treatment. The data were measurement data, which were presented as mean ± standard deviation. The data in two groups were compared using unpaired *t* test, and the data in multiple groups were compared using one‐way ANOVA and Tukey's post hoc test. *P < .05 when compared with treatment of oe‐NC. ^&^ *P* < .05 when compared with treatment of oe‐NC/oe‐YTHDF3/oe‐ITGA6 + mimic NC

A previous study has shown that miR‐448 could inhibit the self‐renewal of HCC cells and thus suppress the occurrence of HCC.^18^ It was also confirmed in the above‐mentioned experiments that miR‐448 could target YTHDF3 to regulate the function of ITGA6. In order to further determine the role of miR‐448/YTHDF3/ITGA6 axis in HCC, we constructed Hep3B cell lines overexpressing YTHDF3 and ITGA6, and then used RT‐qPCR to measure the expression of YTHDF3 and ITGA6 in each group of cells. The results showed that treatment with oe‐YTHDF3 significantly elevated YTHDF3 expression, whereas oe‐ITGA6‐3 significantly increased ITGA6 expression (Figure 4A). Next, oe‐YTHDF3‐, oe‐ITGA6‐ and oe‐NC‐treated Hep3B cells were transfected with miR‐448 mimic and mimic NC, respectively. The results of spheroid formation assay (Figure 4B) and agarose colony formation assay (Figure 4C) manifested that, in the presence of mimic NC, the spheroid formation and colony formation of Hep3B cells were augmented by overexpressing YTHDF3 or ITGA6, which was normalized after additional miR‐448 mimic treatment. Furthermore, as reflected by scratch test (Figure 4D) and Transwell assay (Figure 4E), overexpression of YTHDF3 or ITGA6 enhanced migrating and invasive abilities of cells in the presence of mimic NC, which were neutralized by miR‐448 mimic. We repeated the above experiments using the HCC cell line SMMC7721 (Figure S2A‐E), finding results similar to those in the Hep3B cell line. These results indicate that miR‐448 inhibits spheroid formation, colony formation, invasion and migration of HCC cells by down‐regulating YTHDF3/ITGA6.

### 3.5 KDM5B activated YTHDF3/ITGA6 axis through miR‐448 to promote HCC

**FIGURE 5:**
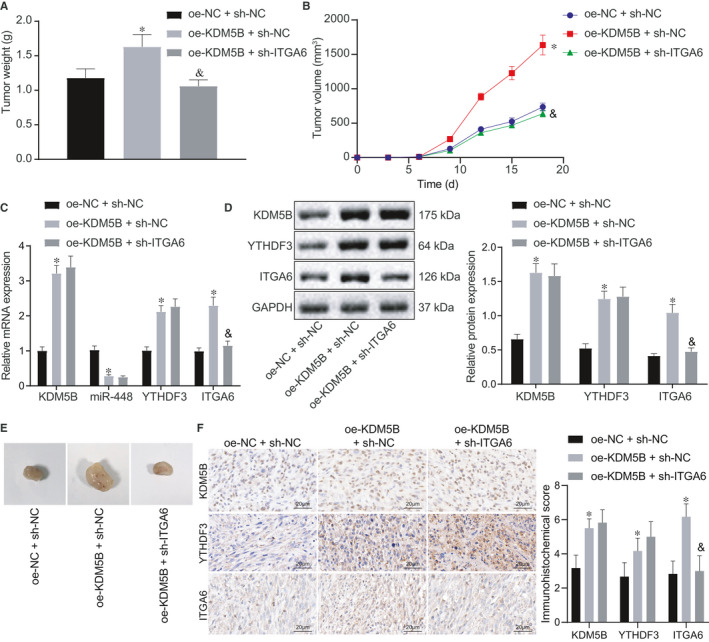
KDM5B activates the YTHDF3/ITGA6 axis through suppressing miR‐448 to promote HCC progression. Cells transfected with oe‐NC + sh‐NC, oe‐KDM5B + sh‐NC or oe‐KDM5B + sh‐ITGA6 were subcutaneously planted into nude mice. A, Weight of xenograft tumours after different treatments. B, Growth curve of subcutaneous xenograft tumours after different treatments. C, RT‐qPCR determination of KDM5B, miR‐448, YTHDF3 and ITGA6 expression in tumour tissues after different treatments. D, Western blot analysis of KDM5B, YTHDF3 and ITGA6 expression in tumour tissues normalized to GAPDH after different treatments. E, Representative images showing xenograft tumours in nude mice. F, Immunohistochemistry of KDM5B, YTHDF3 and ITGA6 proteins in tumour tissues (500×). The data were measurement data, which were presented as mean ± standard deviation. The data in multiple groups were compared using one‐way ANOVA and Tukey's post hoc test. The data in groups at different time were analysed by repeated measures ANOVA followed by Bonferroni's post hoc test. *P < .05 when compared with treatment of oe‐NC + sh‐NC. ^&^ *P* < .05 when compared with treatment of oe‐KDM5B + sh‐NC

Through a series of experiments described above, we demonstrated that KDM5B could target miR‐448 at the cellular level, and miR‐448 inhibited HCC by targeting the YTHDF3/ITGA6 axis. For further verification, we performed a xenograft tumour formation assay in nude mice. Cells transfected with oe‐NC + sh‐NC, oe‐KDM5B + sh‐NC or oe‐KDM5B + sh‐ITGA6 were subcutaneously implanted into nude mice. The results showed that up‐regulation of KDM5B increased the size and weight of tumours and accelerated their growth, which was neutralized by oe‐KDM5B + sh‐ITGA6 treatment (Figure 5A,B). Besides, the expression of KDM5B, miR‐448, YTHDF3 and ITGA6 in mouse tumour tissues was measured by RT‐qPCR; the results revealed that up‐regulation of KDM5B reduced miR‐448 expression, while up‐regulating KDM5B, YTHDF3 and ITGA6 expression. However, overexpression of KDM5B and silencing of ITGA6 together resulted in decreased ITGA6 expression (Figure 5C), which was also confirmed by Western blot analysis and immunohistochemistry (Figure 5D‐F). Collectively, KDM5B could activate the YTHDF3/ITGA6 pathway to enhance the proliferation and growth of tumour cells by inhibiting the expression of miR‐448 in HCC, eventually leading to the occurrence of HCC.

### 3.6 KDM5B inhibitor GSK‐467 depressed hepatocarcinogenesis in vitro and in vivo

**FIGURE 6:**
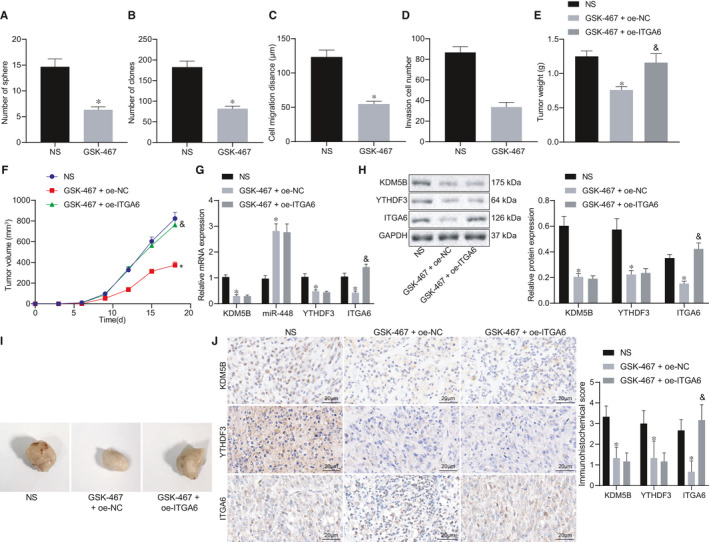
KDM5B inhibitor GSK‐467 blocks HCC progression in vitro and in vivo. A, Cell sphere formation evaluated by cell sphere formation assay. B, Colony formation ability evaluated by clonogenic assay. C, Cell migration detected by scratch test. D, Cell invasion detected by Transwell assay. E, Weight of transplanted tumours. F, Growth curve of subcutaneous transplanted tumours. G, RT‐qPCR to detect the expression of KDM5B, miR‐448, YTHDF3 and ITGA6 in tumour tissues. H, Western blot analysis to detect the expression of KDM5B, YTHDF3 and ITGA6 in tumor tissues. I, Representative images showing xenografts in nude mice. J, Immunohistochemistry of KDM5B, YTHDF3 and ITGA6 proteins in tumour tissues (500×). The data in multiple groups were compared using one‐way ANOVA and Tukey's post hoc test. The data in groups at different time were analysed by repeated measures ANOVA followed by Bonferroni's post hoc test. *P < .05 when compared with the NS group. ^&^ *P* < .05 when compared with the GSK‐467 + oe‐NC group

We purchased GSK‐467 to test its effect on oncogenesis of HCC in vitro and in vivo. To investigate the effect of GSK‐467 on the proliferation ability of HCC cells, HCC cells were treated with normal saline or GSK‐467 for 48 hours, respectively. Cell sphere formation assay, scratch test and Transwell assay showed that the ability of cell sphere formation, clone formation, migration and invasion was weakened in GSK‐467–treated cells relative to normal saline–treated cells (Figure 6A‐D). The above results showed that GSK‐467 could inhibit spheroid formation, colony formation, invasion and migration of HCC cells.

To further validate these results, we monitored xenograft tumour formation in nude mice subcutaneously implanted with transfected cells and assigned into three experimental groups: the NS, the GSK‐467 + oe‐NC and the GSK‐467 + oe‐ITGA6 groups. Results showed that nude mice treated with GSK‐467 had smaller tumours and slower tumour growth rate, which was annulled by oe‐ITGA6 (Figure 6E,F). RT‐qPCR results showed that miR‐448 expression in mice treated with GSK‐467 was increased, whereas KDM5B, YTHDF3 and ITGA6 expression was decreased. Upon treatment with GSK‐467, ITGA6 expression in mice was augmented by oe‐ITGA6 treatment (Figure 6G). Western blot analysis and immunohistochemistry results also confirmed this result at the protein level (Figure 6H‐J). The above results indicated that GSK‐467 could inhibit the proliferation and growth of HCC tumour cells by promoting the expression of miR‐448 and inhibiting the YTHDF3/ITGA6 pathway.

## 4 DISCUSSION

**FIGURE 7:**
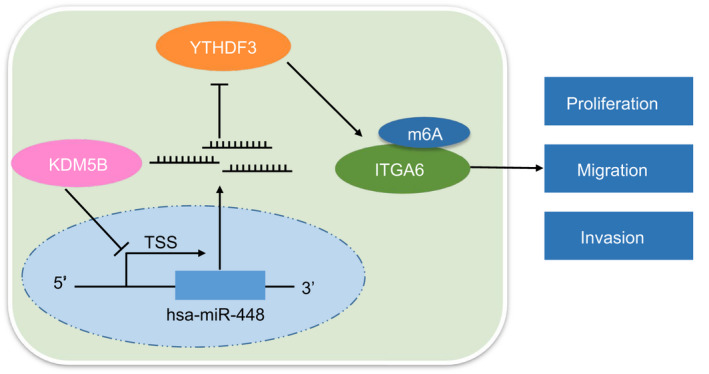
Molecular mechanism of demethylase KDM5B in the occurrence of HCC. KDM5B inhibits miR‐448 expression, and miR‐448 inhibits YTHDF3/ITGA6 axis, eventually leading to accelerated self‐renewal of HCC cells

HCC is a major cause of the cancer‐induced deaths and poses an increasing burden to many areas around the globe, especially in countries located in Africa and Asia where medical resources are limited.^3^ As HCC patients are often diagnosed at the advanced stage, the prognosis is dismal,^8^ which urges scientists to explore novel therapeutic targets to improve the survival of patients with HCC. The histone demethylase KDM5B is highly expressed in several cancers including lung, stomach, breast and hepatic cancers,^11^ making it a potential therapeutic target for HCC. Hence, we experimentally confirmed the possible oncogenic function of KDM5B in HCC and found that KDM5B regulates the YTHDF3/ITGA6 axis by inhibiting the expression of miR‐448 to promote the occurrence of HCC (Figure 7).

Initially, we determined that silencing of KDM5B hindered the self‐renewal, invasion, migration and proliferation of HCC cells. The demethylase KDM5B is known as a potential therapeutic target for cancer treatment, based on its action as a key regulator in important biological processes of cancers such as tumorigenesis, progression and antibiotic resistance, and based on its ability to block the activation of H3K4me3.^19^ KDM5B was found previously to be highly expressed in HCC tissues, and its up‐regulation could accelerate the growth of Hep3B cells and serve as a prognostic marker for HCC.^11^ Moreover, poor expression of KDM5B correlates with better outcome in HCC.^20^ By arresting the cell cycle at the G1/S phase through up‐regulation of p15 and p27, KDM5B inhibits HCC cell proliferation both in vivo and in vitro.^9^ In addition, a prior study elaborated that KDM5B depletion caused reduction in expression of self‐renewal–related genes in trophoblast stem cells, which was partially coincided with our results.^21^ Also, another work uncovered that KDM5B silencing contributed to repression of gastric cancer cell proliferation, migration and invasion in vitro.^22^ These works partially supported our results about the oncogenic role of KDM5B in HCC.

In addition, we found that miR‐448 expression had low expression in HCC tissues and that KDM5B could demethylate H3K4me3 to decrease miR‐448 expression. KDM5B can contribute to tumour cell proliferation through epigenetic repression of tumour suppressor miRs by binding to their promoter regions and by removing the H3K4me3 histone modification associated with transcriptional activation.^16^ Partly in line with our findings, Bamodu et al reported that KDM5B accelerates breast cancer progression and that its high expression was associated with dismal prognosis of breast cancer patients, which could be rescued by treatment with hsa‐miR‐448,^10^ implying an interaction between KDM5B and miR‐448 in promoting tumour growth. Additionally, KDM5B has been found to bind directly to the miR‐448 promoter region and thus suppress its expression in papillary thyroid cancer cells; KDM5B knockdown induced up‐regulation of miR‐448, which abolished the promoting effect of KDM5B on the malignant characteristics of cancer cells.^13^ Up‐regulation of miR‐448 suppresses the progression of non–small‐cell lung cancer by inhibiting cell proliferation and metastasis,^23^ which demonstrates the anti‐cancer function of miR‐448. Furthermore, miR‐448 is able to inhibit the self‐renewal of HCC stem cells and leads to the subsequent suppression of HCC progression through activation of the AMP‐activated protein kinase (AMPK) signalling pathway and down‐regulation of melanoma‐associated antigen 6.^18^ Based on this, KDM5B‐mediated miR‐448 up‐regulation may provide a novel target for the treatment of HCC.

It has been well‐established that miRNAs can interact with the 3′UTR of specific target mRNAs and consequently inhibit their expression.^24^ In this study, the biological prediction website and luciferase reporter assay results identified that miR‐448 bound to the 3′UTR of YTHDF3 mRNA and could negatively regulate its expression. Furthermore, our findings indicated a positive correlation between YTHDF3 and ITGA6 expressions in HCC tissues. Additionally, inactivation of the YTHDF3/ITGA6 axis inhibited the self‐renewal of HCC cells. Existing literature has shown that YTHDF3 is highly expressed in HCC tissues and may result in human hepatocarcinogenesis.^15^ YTHDF3, as one of ‘readers’ of m6A RNA modification, can recognize residues of m6A in ITGA6 and increase ITGA6 expression to promote bladder cancer occurrence.^25^ ITGA6 is also reported to be highly expressed in HCC,^17^ and this overexpression could promote the invasive and metastatic phenotypes of breast cancer stem cells,^26^ suggesting its oncogenic role in HCC. Studies have shown the miRNA regulation of ITGA6. For example, as reported by Guo et al,^27^ miR‐143‐3p can target ITGA6 and reduce its expression, causing an impairment of the metastasis of colorectal cancer. Besides, a binding site has been confirmed between miR‐377‐3p and the 3′UTR of ITGA6 mRNA, indicating that miR‐377‐3p could inhibit ITGA6 expression.^28^ Collectively, miR‐448–mediated YTHDF3/ITGA6 suppression may have the potential to attenuate HCC progression.

Taken together, results of our study corroborate that KDM5B could restrain miR‐448 expression to activate the YTHDF3/ITGA6 axis, thus contributing to HCC progression. This study suggests a promising KDM5B‐targeted therapy for HCC patients and also proposes that a KDM5B/miR‐448/YTHDF3/ITGA6 axis is involved in the occurrence and development of this cancer, which could constitute an important tumour‐related molecular mechanism. Hence, KDM5B inhibitors or other targeted therapies based on this axis are candidates for experimental treatment of human HCC. Moreover, there is increasing evidence that KDM5B silences the AMPK pathway whereas miR‐448 activates it,^18^, ^29^ and that activation of the AMPK pathway inhibits the malignant phenotypes of lung cancer.^30^ Therefore, we will focus in future studies on the role of different signalling pathways involved in the promotive effect of KDM5B on HCC through miR‐448–mediated effects on the YTHDF3/ITGA6 axis, aiming to identify new therapeutic targets for treating this disease.

## CONFLICTS OF INTEREST

The authors declare no conflicts of interest.

## AUTHOR CONTRIBUTION

**Jun‐Cheng Guo:** Conceptualization (equal); Investigation (equal); Methodology (equal); Writing‐original draft (equal). **Zhuo Liu:** Formal analysis (equal); Investigation (equal); Methodology (equal); Writing‐review & editing (equal). **Yi‐Jun Yang:** Conceptualization (equal); Software (lead); Visualization (lead); Writing‐original draft (equal). **Min Guo:** Data curation (equal); Project administration (lead); Writing‐original draft (equal). **Jian‐Quan Zhang:** Formal analysis (equal); Validation (lead); Writing‐review & editing (equal). **Jin‐Fang Zheng:** Data curation (equal); Resources (lead); Supervision (lead); Writing‐review & editing (equal).

## ETHICAL APPROVAL

All research procedures were conducted with approval of the Ethics Committee of Hainan General Hospital and in line with the Declaration of Helsinki. Ethical agreements were obtained from the donors or their relatives by written informed consent prior to experiments. All animal experiments were approved by the Animal Ethics Committee of Hainan General Hospital. Great efforts were made to minimize the number of animals used in the experiments and their suffering.

## Supporting information

Fig S1

Fig S2

## Data Availability

The authors confirm that the data supporting the findings of this study are available within the article.
