# Supplementary figures and images for: KDM5B promotes self‐renewal of hepatocellular carcinoma cells through the microRNA‐448–mediated YTHDF3/ITGA6 axis
Source: J Cell Mol Med. 2021 Apr 7;25(13):5949–62. doi: 10.1111/jcmm.16342 (PMC8256355; doi:10.1111/jcmm.16342)

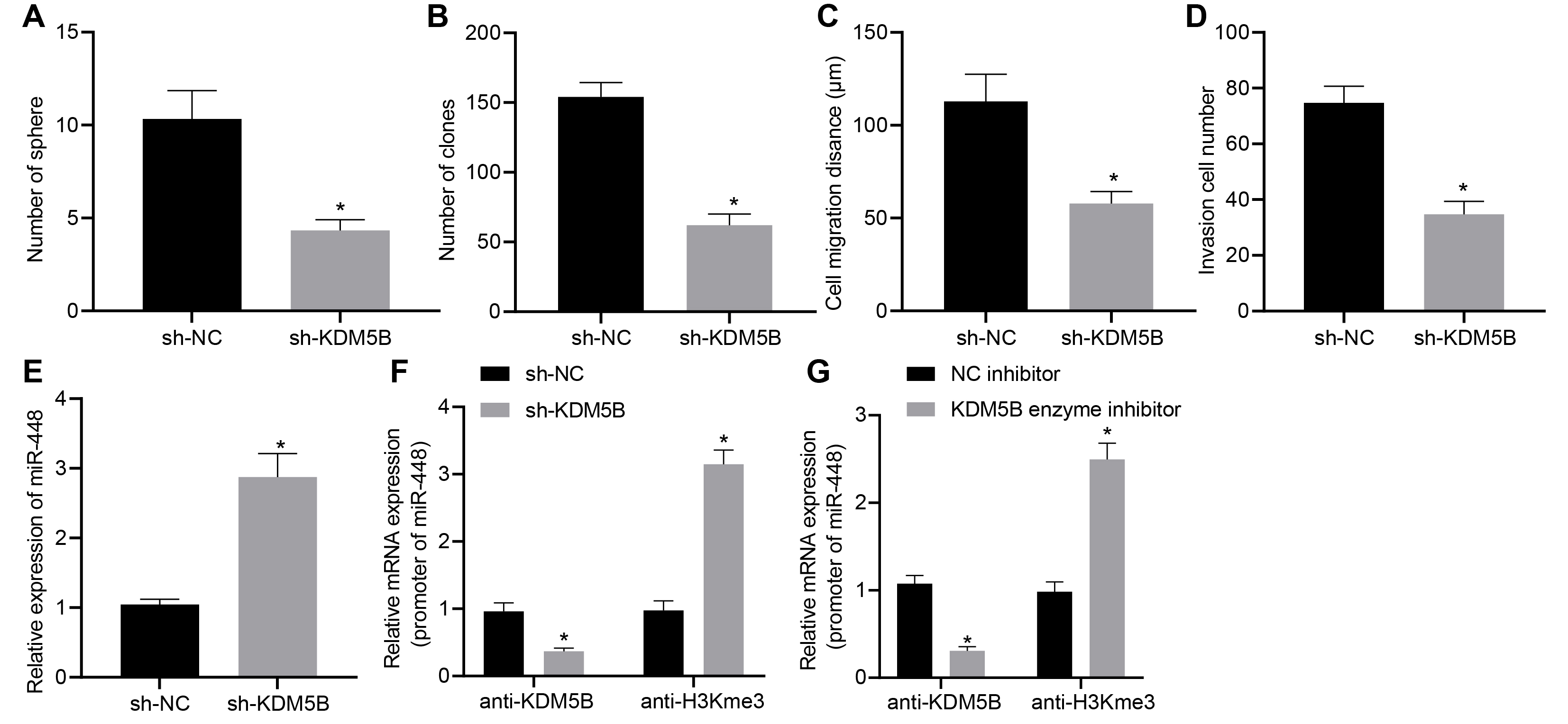

Supplement: Supplementary file 1 — Fig S1 [file JCMM-25-5949-s001.jpg]

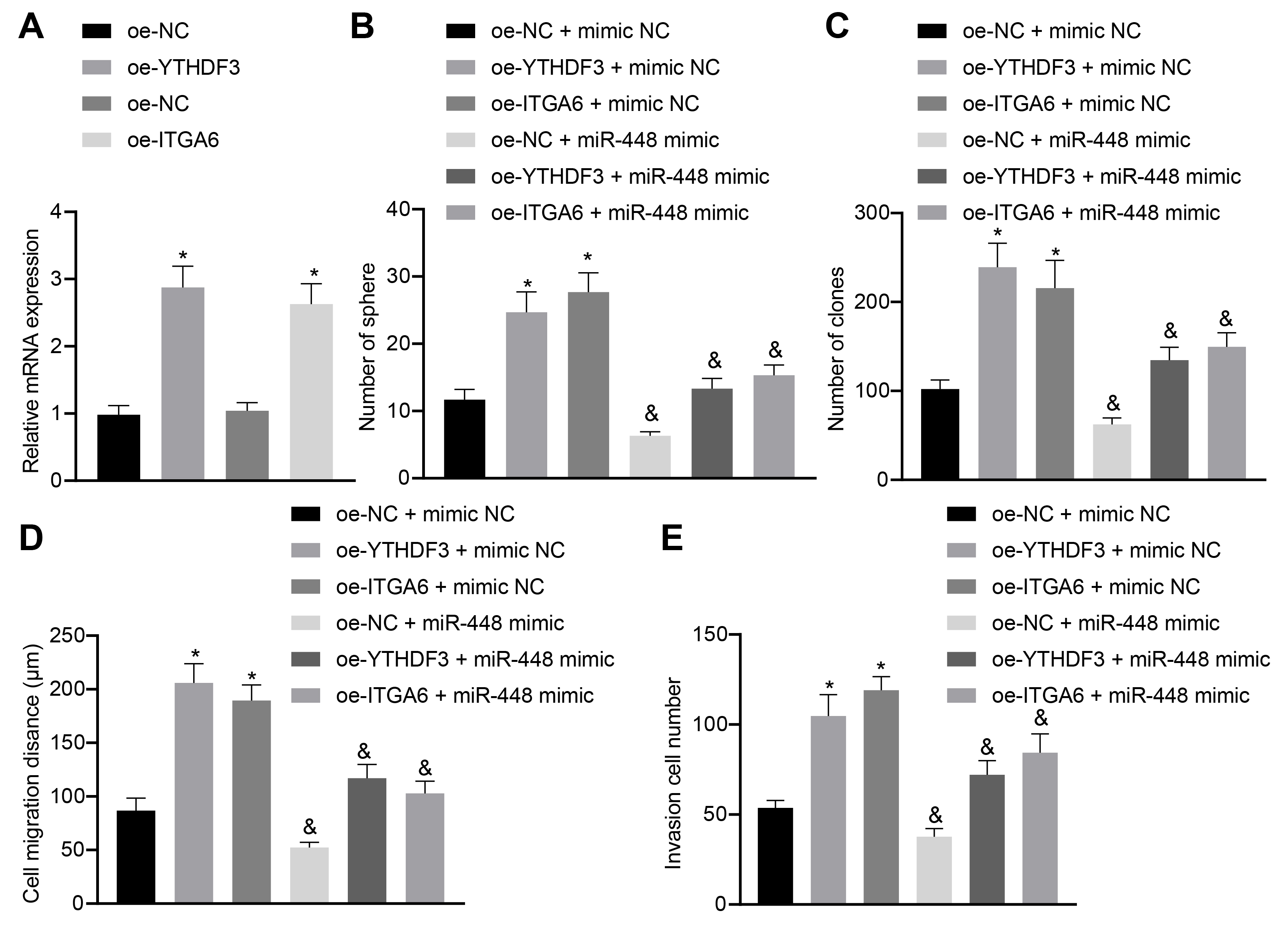

Supplement: Supplementary file 2 — Fig S2 [file JCMM-25-5949-s002.jpg]
